# MiR-98-5p plays suppressive effects on IL-1β-induced chondrocyte injury associated with osteoarthritis by targeting CASP3

**DOI:** 10.1186/s13018-024-04628-9

**Published:** 2024-04-13

**Authors:** Hang Lv, Peiran Liu, Hai Hu, Xiaodong Li, Pengfei Li

**Affiliations:** 1 Department of Orthopedics, Hanan Branch, The Second Affiliated Hospital of Heilongjiang University of Chinese Medicine, No. 411, Guogeli Street, Nangang District, Harbin City, 150060 Heilongjiang Province China; 2 Orthopedic ward, The Third Affiliated Hospital of Heilongjiang University of Chinese Medicine, No. 2 Xiangjiang Road, Xiangfang District, Harbin City, 150000 Heilongjiang Province China

**Keywords:** Osteoarthritis, Chondrocyte, miR-98-5p, CASP3, Extracellular matrix degradation

## Abstract

**Background:**

This study aims to explore how miR-98-5p affects osteoarthritis, focusing on its role in chondrocyte inflammation, apoptosis, and extracellular matrix (ECM) degradation.

**Methods:**

Quantitative real-time PCR was used to measure miR-98-5p and CASP3 mRNA levels in OA cartilage tissues and IL-1β-treated CHON-001 cells. We predicted miR-98-5p and CASP3 binding sites using TargetScan and confirmed them via luciferase reporter assays. Chondrocyte viability was analyzed using CCK-8 assays, while pro-inflammatory cytokines (IL-1β, IL-6, TNF-α) were quantified via ELISA. Caspase-3 activity was examined to assess apoptosis, and Western blotting was conducted for protein marker quantification.

**Results:**

Our results showed lower miR-98-5p levels in both OA cartilage and IL-1β-stimulated cells. Increasing miR-98-5p resulted in reduced pro-inflammatory cytokines, decreased caspase-3 activity, and improved cell viability. Furthermore, miR-98-5p overexpression hindered IL-1β-induced ECM degradation, evident from the decline in MMP-13 and β-catenin levels, and an increase in COL2A1 expression. MiR-98-5p's impact on CASP3 mRNA directly influenced its expression. Mimicking miR-98-5p's effects, CASP3 knockdown also inhibited IL-1β-induced inflammation, apoptosis, and ECM degradation. In contrast, CASP3 overexpression negated the suppressive effects of miR-98-5p.

**Conclusions:**

In conclusion, our data collectively suggest that miR-98-5p plays a protective role against IL-1β-induced damage in chondrocytes by targeting CASP3, highlighting its potential as a therapeutic target for OA.

## Background

Osteoarthritis (OA) is the most common chronic joint disease, marked by joint inflammation and progressive articular cartilage degeneration [1]. It is projected that by 2050, about 130 million people worldwide will suffer from OA, with nearly 40 million of these cases evolving into severe disabilities [2]. Chondrocytes, the sole cell type in articular cartilage, are crucial for maintaining tissue balance and matrix integrity [3, 4]. The pathogenesis of OA is strongly linked to reduced chondrocyte viability and extracellular matrix (ECM) degradation, often triggered by cytokines and growth factors [5, 6]. Therefore, understanding the molecular processes that disrupt chondrocytes is key to developing effective treatments for OA.

Recent studies have highlighted the role of non-coding RNAs, including small interfering RNAs (siRNAs) [7] and microRNAs (miRNAs) [8, 9], in musculoskeletal disorders. MicroRNAs, small (~ 22 nucleotides) endogenous non-coding RNAs, regulate gene expression at transcriptional and post-transcriptional levels by binding to the 3′-untranslated regions (3′-UTRs) of target mRNAs [10]. Notably, the dysregulation of several miRNAs has been linked to cartilage degradation and OA progression [11–13]. For instance, miR-296-5p was found to promote chondrocyte proliferation and inhibit apoptosis and matrix-degrading enzyme expression in response to IL-1β by targeting TGF-β1 [14]. MiR-9, upregulated in OA rat cartilage, was shown to combat OA by reducing ECM degradation [15]. Additionally, elevated miR-203a levels in OA tissues and models have been suggested to contribute to cartilage degradation by targeting Smad3 [16]. MiR-98-5p, known for its dysregulation in inflammatory diseases such as ulcerative colitis [17], acute myocardial infarction [18], and asthma [19], has also been implicated in various biological processes. For example, it was involved in the injury of ox-LDL-induced HUVEC cells as a downstream gene of circ-USP36 [20], protected against cerebral ischemia/reperfusion injury [21], and was shown to impact bone regeneration by affecting osteogenic differentiation and osteoblast growth [22]. Intriguingly, Huang et al. [23] identified miR-98-5p as a key miRNA in OA progression through bioinformatics analysis.

Caspases, a family of cysteine proteases comprising 15 members, are critical in programmed cell death and inflammation [24]. Of these, caspase-3 (CASP3) is a prominent executor in the apoptotic process, extensively researched in relation to cancer development and therapy [25–27]. CASP3's role in OA pathogenesis is also well-documented. For instance, Zou et al. [28] demonstrated that genistein's anti-apoptotic effects on chondrocytes, including reduced CASP3 expression, contribute to decreased inflammation and lessened cartilage degradation in OA treatment. High glucose levels over long periods have been found to increase CASP3 expression, leading to chondrocyte apoptosis and cytoskeleton aggregation [29]. Thomas et al. [30] also highlighted that apoptosis-induced chondrocyte death could impact cartilage metabolism, underlining its significance in OA pathogenesis. Our previous studies align with findings by Huang et al. [23], suggesting CASP3 as a potential target of miR-98-5p. This leads to the hypothesis that miR-98-5p may mitigate OA by modulating chondrocyte inflammation, apoptosis, and ECM degradation through targeting CASP3.

In this study, we measured the expression of miR-98-5p and CASP3 in OA patient cartilage samples. Using miRNA target prediction, we verified the link between miR-98-5p and CASP3 in the IL-1β-stimulated chondrocyte cell line CHON-001. Our functional experiments focused on whether miR-98-5p influences IL-1β-induced inflammation, apoptosis, and ECM degradation in CHON-001 cells by targeting CASP3.

## Materials and methods

### Clinical specimen collection

The blood and cartilage tissues from the femoral condyle and tibial plateau were obtained from twenty patients undergo total knee replacement for end-stage knee OA (13 males and 7 females, with an average age of 51.3 years old). Normal human articular cartilage without arthritis was collected from the knee or hip joints from 20 patients with osteosarcoma or trauma who were undergoing surgery (12 males and 8 females, with an average age of 45.6 years old) as normal control. For the current study, we specifically selected participants who primarily exhibited medial knee OA, as indicated by a medial joint space width (JSW) narrower than the lateral JSW based on radiographic evaluations. This approach aligns with the guidelines recommended by the Osteoarthritis Research Society International [31]. Blood samples at baseline knee OA were centrifuged to extract serum for ELISA assay. Simultaneously, knee cartilage tissues were immediately preserved in liquid nitrogen for subsequent analysis. Informed consent was secured from all participating patients. This study adhered to the Declaration of Helsinki guidelines and received approval from the Ethics Committee of The Second Affiliated Hospital of Heilongjiang University of Chinese Medicine (Heilongjiang Province, China).

### Cell culture and treatment

Human chondrocyte cell line CHON-001 derived from normal articular cartilage was obtained from American Type Culture Collection (ATCC, Manassas, VA, USA). CHON-001 cells were grown in DMEM (Gibco, Grand Island, USA) with 10% FBS (Gibco) at 37 °C with CO_2_. Then, OA cellular model in vitro was established in CHON-001 cells by 24 h stimulation with 10 ng/mL IL-1β [32, 33] (Sigma Aldrich, St. Louis, MO, USA).

### Cell transfection

The miR-98-5p mimics (5′-UGAGGUAGUAAGUUGUAUUGUU-3′), miRNA negative control (miR-NC: 5ʹ-ACCAUGGCUGUAGACUGUUAUU-3′), small interfering RNA (siRNA) against CASP3 (si-CASP3: 5ʹ-GGAAGCGAATCAATGGACTCT-3′), siRNA negative control (si-NC: 5ʹ-GACGAACTGATGCGCTAAGAT-3′), pcDNA3.1-based CASP3 overexpression vector (CASP3) and pcDNA3.1 empty vector were synthesized by GenePharma Co Ltd (Shanghai, China). CHON-001 cells were seeded into plates with six wells (2.0 × 10^5^ per well) and cultured overnight to reach 80% cell density. In accordance with the instructions of Lipofectamine 2000 (Invitrogen, Carlsbad, USA), CHON-001 cells were transfected with 40 nM oligos or 1 μg vectors for 48 h, followed by 24 h stimulation with 10 ng/mL IL-1β.

### Enzyme-linked immunosorbent assay (ELISA) assay

The serum was diluted at a 1:100 ratio and the supernatant from CHON-001 cells at a 1:50 ratio for the assessment of inflammation. The levels of interleukins IL-1β and IL-6, along with TNF-α, were quantified using specific ELISA kits (RayBiotech, Peachtree Corners, GA, USA). Three biological replicates were performed for the statistical analysis.

### Quantitative real-time PCR

Total RNA was isolated using TRIzol Reagent (Thermo Fisher Scientific, Waltham, MA, USA) as per the manufacturer's instructions, including RNA extraction with TRIzol and chloroform, isopropanol precipitation, ethanol wash, drying, and resuspension of the RNA pellet in nuclease-free water. Reverse transcription was performed with TaqMan MicroRNA Reverse Transcription Kit (Applied Biosystems, Foster City, CA, USA) or PrimeScript RT reagent kit (Applied Biosystems). The expression of miR-98-5p and CASP3 mRNA was quantified with TaqMan miRNA assay (Takara, Shiga, Japan) and SYBR Premix Ex Taq (Takara), respectively on a CFX Connection Real-Time System (Bio-Rad). The cycling conditions were as follows: 30 s at 95 °C, followed by 40 cycles of 95 °C for 5 s and 60 °C for 30 s. Relative gene expression level was calculated using 2^−ΔΔCt^ method [34]. The primers used were listed as follows: miR-98-5p forward: 5′-ATCCAGTGCGTGTCGTG-3′ and reverse: 5′-TGCTTGAGGTAGTAAGTTG-3′; U6 forward: 5′-CTCGCTTCGGCAGCACA-3′ and reverse: 5′-AACGCTTCACGAATTTGCGT-3′; CASP3 forward: 5′-TTGGAACCAAAGATCATACATGGAA-3′ and reverse: 5′-TGAGGTTTGCTGCATCGACA-3′; GAPDH forward: 5′-GGTGAAGGTCGGAGTCAACG-3′ and reverse: 5′-GCATCGCCCCACTTGATTTT-3′. Three biological replicates were performed for the statistical analysis.

### Cell viability assay

We performed cell counting kit-8 (CCK-8) assay to analyze the chondrocyte viability. In brief, cells were seeded into 96-well plates at a density of 4,000 cells per well. After culture for 24, 48 and 72 h, we discarded the supernatant and added 10 µl of CCK-8 solution (Beyotime, Shanghai, China) to each well. Following 2 h incubation at 37 °C, the absorbance at 450 nm was measured with MultiMode Microplate Reader (Thermo Fisher Scientific). Three biological replicates were performed for the statistical analysis.

### Caspase-3 activity assay

To evaluate cell apoptosis, the caspase-3 activity was determined using a caspase-3 colorimetric assay kit (Abcam, Cambridge, UK) according to the instructions of manufacturer. Using a microplate reader (BioTek, Winooski, VT, USA), we measured the optical density value at 400 nm and normalized relative caspase-3 activity to the control group. Three biological replicates were performed for the statistical analysis.

### Western blot

The protein samples of CHON-001 cells were extracted by radio-immunoprecipitation assay buffer (RIPA, Beyotime, China) and concentration was examined by a BCA protein assay Kit (Beyotime). Equal amount of protein (30 μg) was isolated by 10% SDS-PAGE and transferred into PVDF membranes. After performing 2 h blocking with 5% non-fat milk at room temperature, the membranes were probed with primary antibodies against caspase-3, Bcl-2, Bax, COL2A1, MMP-13, β-catenin and GAPDH (Abcam Cambridge, MA, USA) overnight at 4 °C. Subsequently, the membranes were incubated with the secondary antibody conjugated by horseradish peroxidase for 2 h at room temperature and then examined using enhanced chemiluminescence solution (Bio-Rad, Hercules, USA). Three biological replicates were performed for the statistical analysis.

### Target prediction and luciferase reporter assay

The potential interaction between miR-98-5p and CASP3 was analyzed using TargetScan 7.1 (http://www.targetscan.org/). For the luciferase reporter assay, we created both wild-type (WT) and mutant (MUT) CASP3 luciferase reporter vectors. This involved inserting CASP3 3′-UTR sequences with either miR-98-5p binding or mutant sites into pmirGLO vectors (Promega, Madison, WI, USA). We then co-transfected CHON-001 cells with 0.2 µg of either WT or MUT CASP3 vectors and 20 nM miR-98-5p mimics or miR-NC. The cells were plated in 24-well plates at a density of 4.0 × 10^5^ cells per well and transfected using Lipofectamine 2000 (Invitrogen). After 48 h incubation, we measured Firefly and Renilla luciferase activities using a Dual Luciferase Reporter Assay Kit (Promega), and the Firefly/Renilla luciferase ratio was used to determine relative luciferase activity. Three biological replicates were performed for the statistical analysis.

### Statistical analysis

Data processing was performing with GraphPad Prism 6.0 (GraphPad Software, La Jolla, CA). All data were presented as the mean ± standard deviation (SD) from three independent biological replications. Statistical differences between two groups were assessed by Student’s t-test and that among multiple groups were evaluated by one-way analysis of variance (ANOVA) followed by Turkey’s post hoc test. All *p*-values less than 0.05 were deemed statistically significant.

## Results

### Decreased miR-98-5p and increased CASP3 in OA cartilage and cellular models

**Fig. 1:**
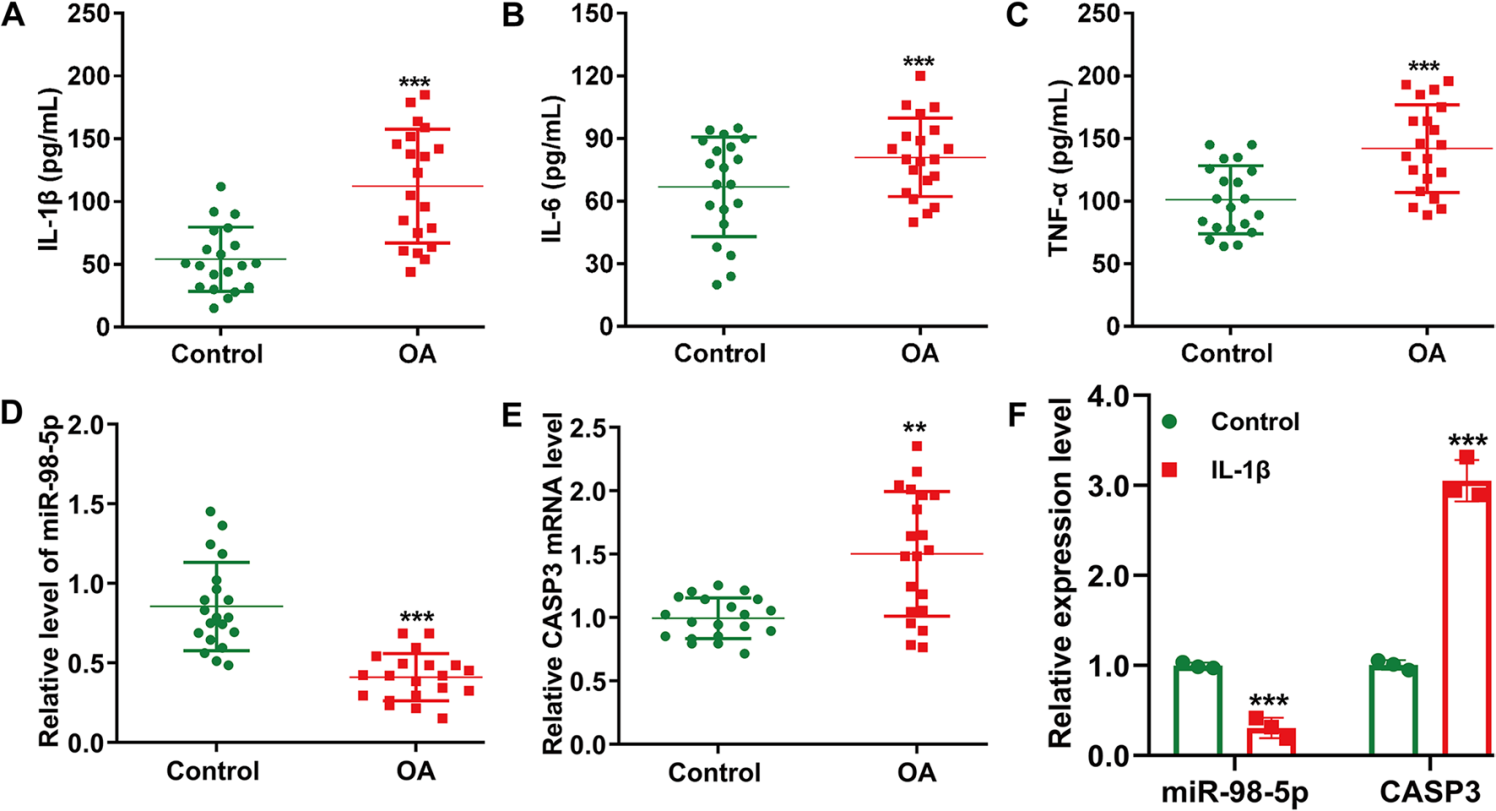
Expression levels of miR-98-5p and CASP3 in OA cartilage and OA cellular model. (**A-C**) ELISA assay was applied to determine the levels of IL-1β, IL-6 and TNF-α in OA-affected blood samples (n = 20) and control samples (n = 20). Quantitative real time PCR was used to detect the expression levels of miR-98-5p (**D**) and CASP3 mRNA (**E**) in cartilage samples of OA patients (n = 20) and control samples (n = 20). **p < 0.01 and ***p < 0.001 indicate OA versus Control; (**F**) Quantitative real time PCR was used to detect the expression levels of miR-98-5p and CASP3 mRNA in IL-1β-induced CHON-001 cells and controls. ***p < 0.001 indicates IL-1β versus Control

Initially, we gathered blood samples from 20 primary patients at baseline knee OA and 20 control subjects to assess inflammation levels. ELISA results indicated a substantial increase in pro-inflammatory cytokines (IL-1β, IL-6 and TNF-α) in the OA group compared to controls (Fig. 1A–C). We also measured miR-98-5p and CASP3 in OA cartilage tissues using quantitative real-time PCR. Figure 1D demonstrated a marked decrease in miR-98-5p levels in OA patient cartilage compared to controls. Figure 1E shows a significant increase in CASP3 mRNA in OA cartilage versus control tissues. Additionally, we used IL-1β to induce OA in CHON-001 cells in vitro. This resulted in a notable reduction in miR-98-5p and a rise in CASP3 mRNA, as illustrated in Fig. 1F.

### Overexpression of miR-98-5p weakened IL‐1β‐caused inflammatory, apoptosis and ECM degradation in chondrocytes

**Fig. 2:**
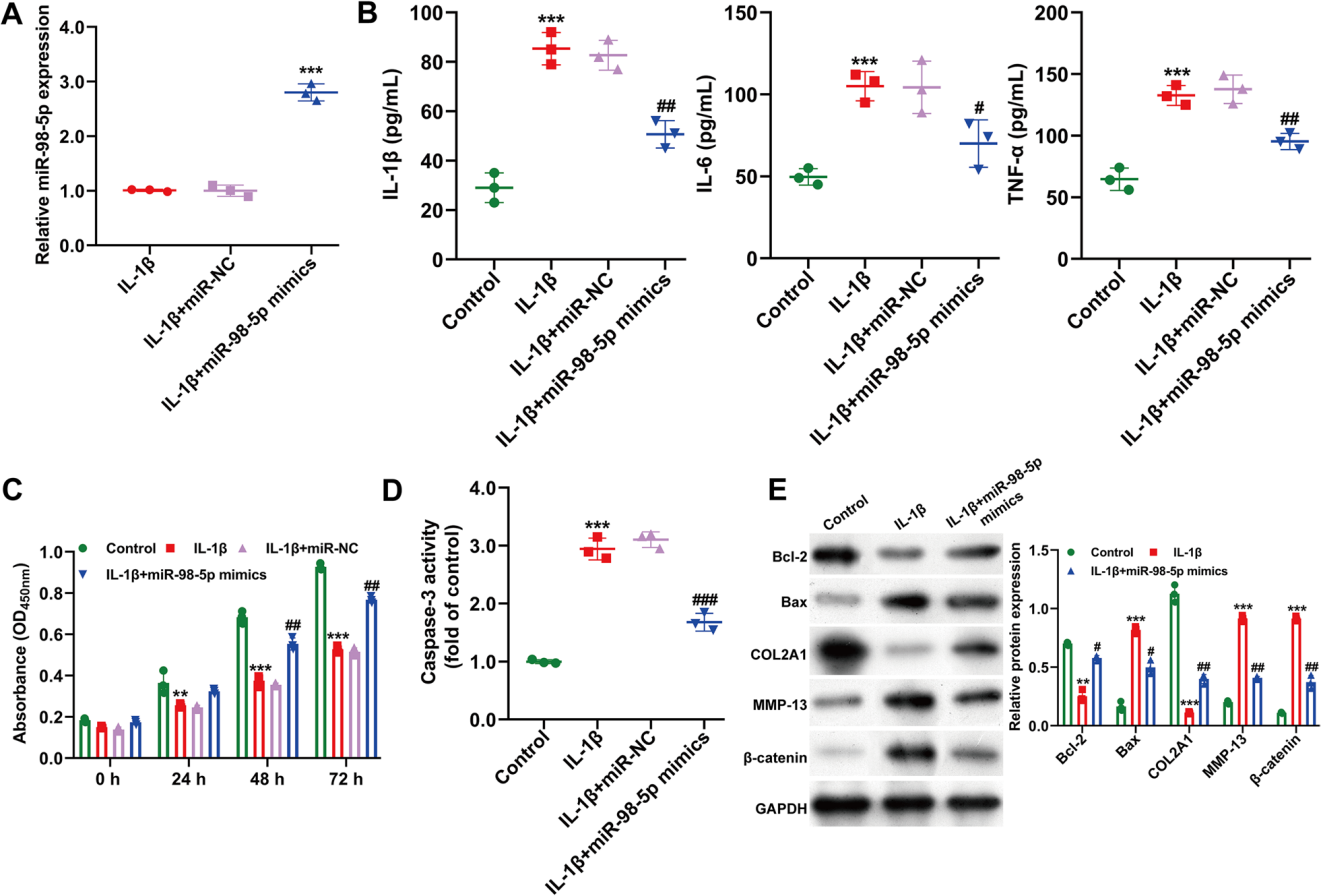
Effects of miR-98-5p overexpression on IL‐1β‐caused inflammatory, apoptosis and ECM degradation in chondrocytes. **A** The expression of miR-98-5p was determined in CHON-001 cells transfected with miR-98-5p mimics or miR-NC after treatment of IL-1β. ***p < 0.001 indicates IL-1β + miR-98-5p mimics versus IL-1β + miR-NC; The inflammatory cytokines (**B**), cell viability (**C**), and caspase-3 activity (**D**) in CHON-001 cells transfected with miR-98-5p mimics or miR-NC after treatment of IL-1β. **p < 0.01 and ***p < 0.001 indicate IL-1β versus Control; #*p* < 0.05, ##*p* < 0.01, ###*p* < 0.001 indicates IL-1β + miR-98-5p mimics versus IL-1β + miR-NC; **E** The protein levels of Bcl-2, Bax, COL2A1, MMP-13 and β-catenin were detected in CHON-001 cells transfected with miR-98-5p mimics or miR-NC after treatment of IL-1β. **p < 0.01 and ***p < 0.001 indicate IL-1β versus Control; #*p* < 0.05 and ##*p* < 0.01 indicate IL-1β + miR-98-5p mimics versus IL-1β. All data are shown as means ± SD of three independent experiments

To explore miR-98-5p's role in chondrocyte damage caused by IL‐1β, we transfected CHON‐001 cells with miR-98-5p mimics or miR-NC before introducing 10 ng/mL of IL‐1β. Figure 2A showed that miR-98-5p mimic transfection significantly increased miR-98-5p levels in IL‐1β-stimulated CHON-001 cells compared to miR-NC. ELISA results revealed that miR-98-5p upregulation reduced IL‐1β-induced IL‐1β, IL‐6, and TNF‐α secretion (Fig. 2B). We then assessed miR-98-5p's impact on cell survival and apoptosis. Figure 2C indicated that miR-98-5p mimics mitigated the reduction in cell viability caused by IL‐1β. Similarly, miR-98-5p's overexpression lessened the increase in caspase-3 activity due to IL‐1β exposure (Fig. 2D). Additionally, western blotting was used to evaluate proteins related to apoptosis and ECM degradation. Figure 2E showed that miR-98-5p overexpression decreased IL‐1β-induced apoptosis by upregulating Bcl-2 and downregulating Bax, and reduced ECM degradation by increasing COL2A1 while decreasing MMP-13 and β-catenin in CHON-001 cells. Overall, these findings suggest that miR-98-5p overexpression mitigates IL‐1β-induced chondrocyte injury.

### CASP3 as a potential target of miR-98-5p

**Fig. 3:**
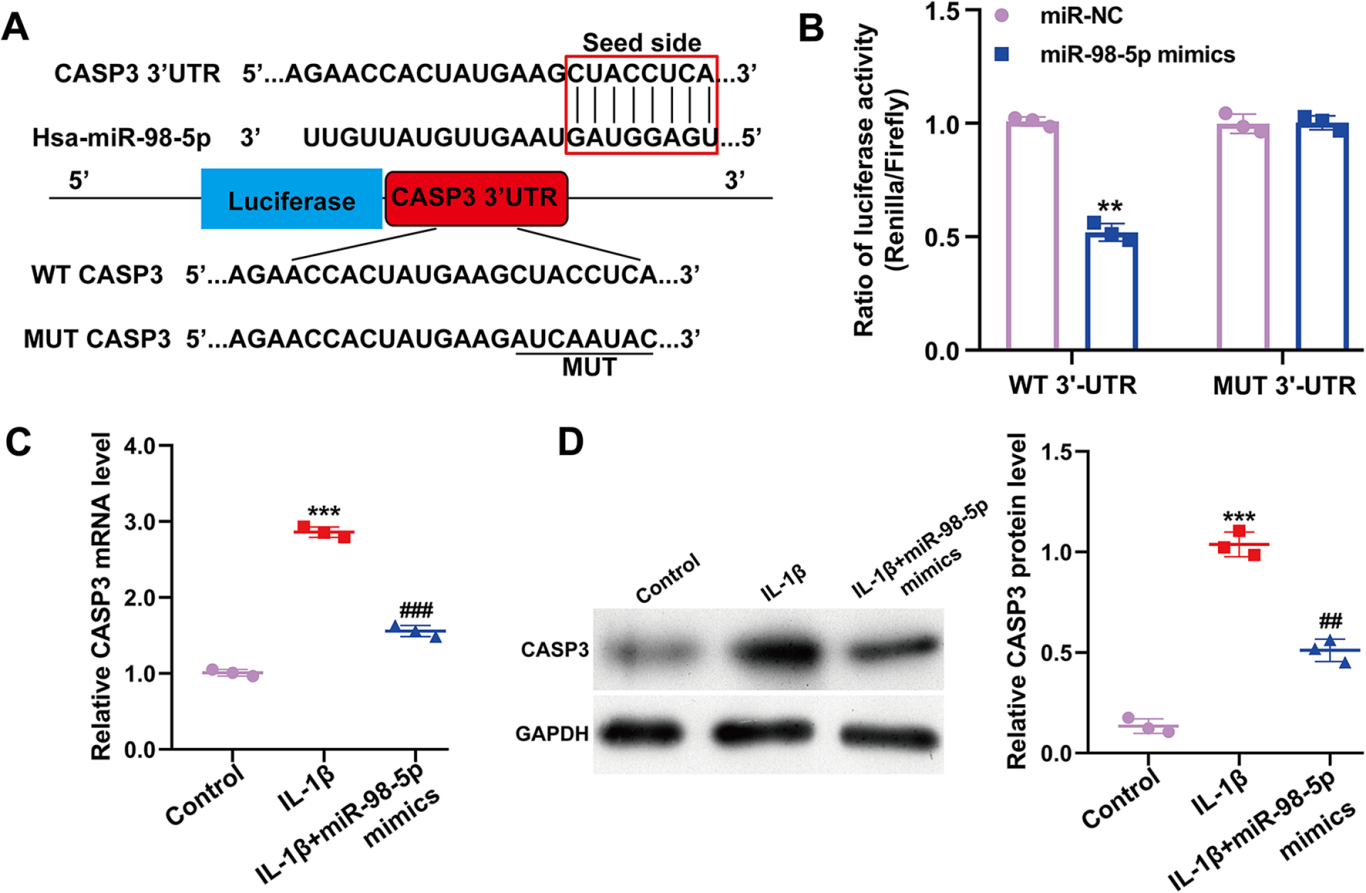
CASP3 as a potential target of miR-98-5p. **A** Sequence alignment of a putative miR-98-5p binding site within the 3′-UTR of CASP3 mRNA was shown. **B** CHON-001 cells were co-transfected with miR-98-5p mimics or miR-NC and luciferase reporter constructs of the wild-type CASP3 3′-UTR (WT 3′-UTR) or the mutated CASP3 3′-UTR (MUT 3′-UTR). Luciferase activity was measured after transfection. **p < 0.01 indicates miR-98-5p mimics versus miR-NC; The CASP3 mRNA (**C**) and protein (**D**) levels were measured in IL-1β-stimulated CHON-001 cells. All data are shown as means ± SD of three independent experiments. ***p < 0.001 indicates IL-1β versus Control; ##*p* < 0.01 and ###*p* < 0.001 indicate IL-1β + miR-98-5p mimics versus IL-1β

Target prediction tools identified the 3′-UTR regions of human CASP3 as potential targets of miR-98-5p, featuring specific seed sequences (Fig. 3A). To confirm miR-98-5p's direct interaction with CASP3 mRNA's 3′-UTR, a luciferase reporter assay was performed. Results depicted in Fig. 3B indicated that cells transfected with miR-98-5p mimics and WT 3′-UTR of CASP3 showed a significant reduction in luciferase activity, unlike cells with the MUT 3′-UTR. Additionally, we observed an increase in both CASP3 mRNA (Fig. 3C) and protein levels (Fig. 3D) in IL‐1β-stimulated CHON-001 cells, which miR-98-5p overexpression notably reduced.

### Knockdown of CASP3 suppressed IL‐1β‐induced inflammatory, apoptosis and ECM degradation in chondrocytes

**Fig. 4:**
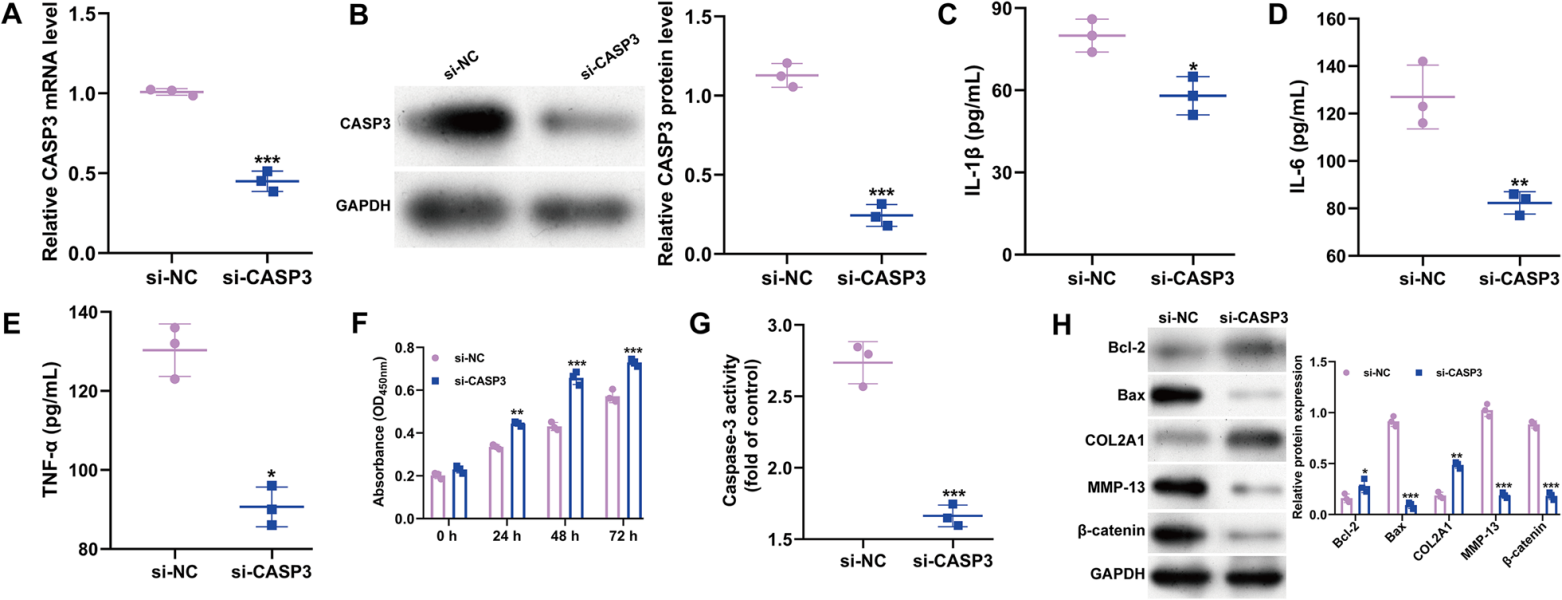
Effects of CASP3 knockdown on IL‐1β‐caused inflammatory, apoptosis and ECM degradation in chondrocytes. CHON-001 cells were transfected with si-CASP3 or si-NC, and then exposed to IL-1β for 24 h. The mRNA (**A**) and protein (**B**) levels of CASP3 were analyzed using quantitative real time PCR and western blot. **C** ELISA assay was applied to determine the production of IL-1β, IL-6 and TNF-α. **D** Cell viability was assessed by CCK-8 assay. (E) Caspase-3 activity was determined in CHON-001 cells. All data are shown as means ± SD of three independent experiments. *p < 0.05, **p < 0.01 and ***p < 0.001 indicate si-CASP3 versus si-NC; (**F**) The protein levels of Bcl-2, Bax, COL2A1, MMP-13 and β-catenin (**E**) were detected in CHON-001 cells

We then investigated CASP3's role in IL‐1β-induced chondrocyte damage. Given CASP3's upregulation in IL-1β-treated chondrocytes, we conducted a loss-of-function study in CHON-001 cells using si-CASP3 transfection. The efficacy of si-CASP3 knockdown was initially verified by quantitative real-time PCR (Fig. 4A) and western blot (Fig. 4B). Following this, ELISA results showed that CASP3 silencing reduced the release of pro-inflammatory cytokines like IL-1β, IL‐6, and TNF‐α (Fig. 4C-E). The CCK-8 assay indicated improved viability in IL-1β-stimulated CHON-001 cells post-CASP3 knockdown (Fig. 4F). si-CASP3 transfection also decreased caspase-3 activity in these cells (Fig. 4G). Furthermore, western blotting for apoptosis and ECM degradation markers revealed that CASP3 knockdown decreased Bax, MMP-13, and β-catenin levels, while increasing Bcl-2 and COL2A1 expression in IL-1β-treated CHON-001 cells (Fig. 4H).

### Upregulation of CASP3 reversed the effects of miR-98-5p on IL-1β-induced inflammatory, apoptosis and ECM degradation in chondrocytes

**Fig. 5:**
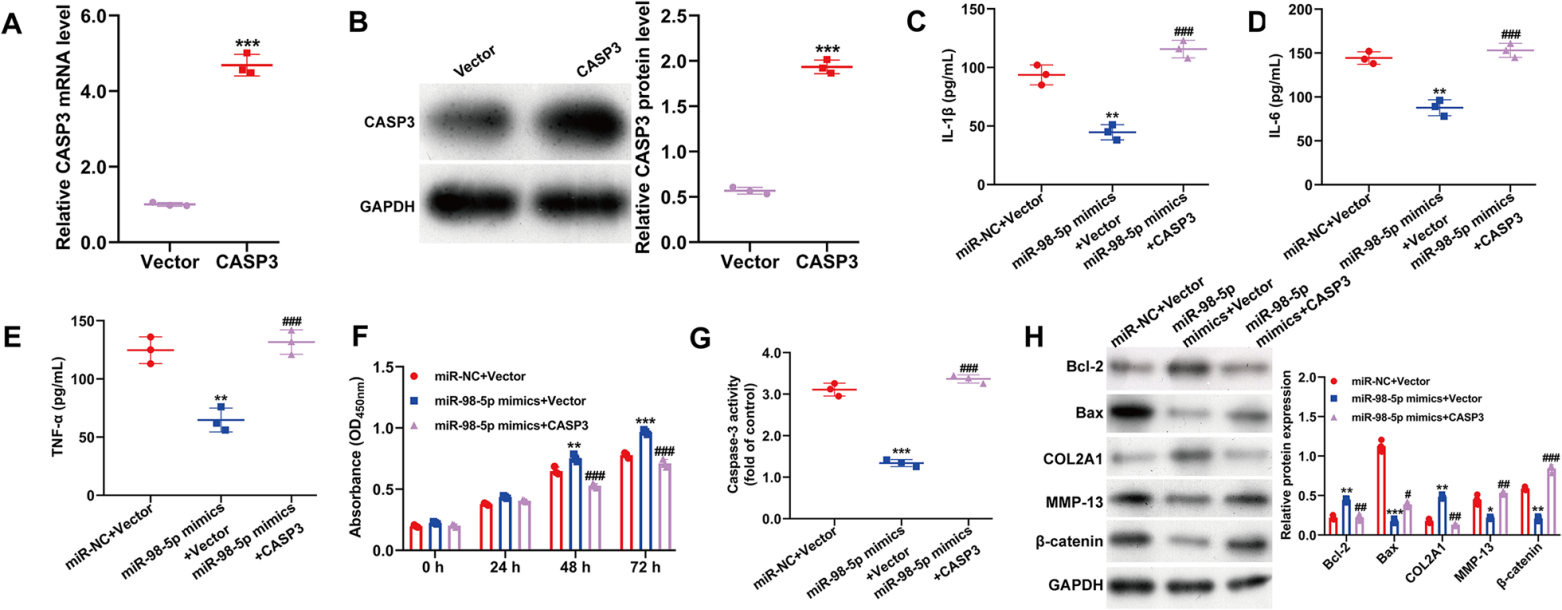
Upregulation of CASP3 reversed the effects of miR-98-5p on IL-1β-induced inflammatory, apoptosis and ECM degradation in chondrocytes. The mRNA (**A**) and protein (**B**) levels of CASP3 were analyzed in CHON-001 cells transfected with CASP3 overexpression plasmid or empty vector, followed by 24 h treatment with IL-1β. ***p < 0.001 indicates CASP3 versus Vector; **C**–**E** Proinflammatory cytokines, **F** cell viability, **G** caspase-3 activity, and **H** protein levels of Bcl-2, Bax, COL2A1, MMP-13 and β-catenin were detected in CHON-001 cells co-transfected with miR-98-5p mimics and CASP3, followed by 24 h treatment with IL-1β. All data are shown as means ± SD of three independent experiments. **p < 0.01 and ***p < 0.001 indicate miR-98-5p mimics + Vector versus miR-NC + Vector; ##*p* < 0.01 and ###*p* < 0.001 indicate miR-98-5p mimics + CASP3 versus miR-98-5p mimics + Vector

To determine if miR-98-5p exerts its effects through CASP3, we conducted rescue experiments in IL-1β-treated CHON-001 cells, co-transfecting them with miR-98-5p mimics and CASP3. Initially, CASP3 overexpression was validated by quantitative real-time PCR (Fig. 5A) and western blot (Fig. 5B). Subsequent functional assays revealed that CASP3 overexpression negated the miR-98-5p-induced decrease in pro-inflammatory cytokines (Fig. 5C–E), increase in cell viability (Fig. 5F), and reduction of caspase-3 activity (Fig. 5G) in IL-1β-treated cells. Moreover, CASP3 re-expression mitigated the impact of miR-98-5p on the protein levels of Bcl-2, Bax, COL2A1, MMP-13, and β-catenin (Fig. 5H).

## Discussion

Growing research highlights the importance of miRNAs in maintaining chondrocyte balance, a key factor in OA development [35]. In our study, we noted a marked decrease in miR-98-5p and an increase in CASP3 in both OA cartilage and IL-1β-treated CHON-001 cells, relative to their respective controls. Functionally, miR-98-5p modulates CASP3, leading to reduced markers of inflammation, apoptosis, and extracellular matrix (ECM) degradation in these cells. These findings underscore miR-98-5p's potential role as a critical regulator in OA onset and progression, aligning with previously reported studies [23].

Inflammation, apoptosis, and cartilage deterioration are key factors in OA progression [36, 37]. Our study using IL-1β-stimulated CHON-001 cells showed enhanced cell viability, reduced caspase-3 activity and pro-inflammatory cytokines (IL-1β, IL-6, TNF-α), and diminished ECM degradation in cells overexpressing miR-98-5p compared to controls. Correspondingly, miR-98 overexpression in isolated human chondrocytes reduced IL-1β-induced TNF-α production [38] and inhibited apoptosis in OA cartilage cells [39, 40] Zheng et al. [22] showed miR-98-5p's role in inhibiting osteogenic differentiation and osteoblast growth by targeting HMGA2. Our study differs from these by using the IL-1β-stimulated CHON-001 cell model to explore miR-98-5p's effect on inflammation, apoptosis, and ECM degradation in OA pathogenesis. This model is validated for representing healthy cartilage's sole cellular component and its association with cartilage damage in OA [41–43].

CASP3 has been recognized as a potential biomarker for OA prognosis in the Equus Asinus model [44]. In our study, we predicted and confirmed CASP3 as a target gene of miR-98-5p in IL-1β-stimulated CHON-001 cells. Aligning with its role in promoting apoptosis, CASP3 knockdown replicated, while its overexpression significantly reversed, miR-98-5p's inhibitory effects on IL-1β-induced pro-inflammatory cytokine release, caspase-3 activity, and ECM degradation in CHON-001 cells. At the molecular level, CASP3 reintroduction moderated miR-98-5p's influence on proteins like Bcl-2, Bax, COL2A1, MMP-13, and β-catenin. The contrast between anti-apoptotic Bcl-2 and pro-apoptotic Bax and CASP3 is notable. COL2A1, a primary component of cartilage matrix, is susceptible to degradation by MMPs, particularly MMP-13 [45]. β-Catenin, crucial in the canonical Wnt signaling pathway, has been linked to cartilage degeneration in OA [46]. Studies show that miR-98 can inhibit the Wnt/β-catenin signaling pathway in hepatocellular carcinoma [47] and retinoblastoma [48], possibly explaining miR-98-5p's role in reducing ECM degradation in IL-1β-stimulated CHON-001 cells. Previous bioinformatics analysis identified the miR-98-5p → CASP3 regulatory pair as relevant to OA pathogenesis [23]. Our research furthers this understanding by detailing how CASP3, as a downstream regulator, plays a role in miR-98-5p's mitigation of IL-1β-induced chondrocyte injury in vitro.

## Conclusions

In conclusion, our research highlights the effects of miR-98-5p on alleviating IL-1β-induced inflammation, apoptosis, and ECM degradation in chondrocytes are mediated through regulation of CASP3. These insights present a promising target and lay a theoretical groundwork for future treatments of human OA.

## Data Availability

All data generated or analyzed during this study are included in this manuscript.
